# The sampling bottleneck in antimicrobial susceptibility testing: consequences and paths forward

**DOI:** 10.1128/jcm.01788-25

**Published:** 2026-04-09

**Authors:** Ivan Brukner, Mathew Oughton

**Affiliations:** 1 Microbiology Laboratory, Jewish General Hospital 5621, Montréal, Québec, Canada; 2 Department of Medicine, McGill University 5620, Montréal, Québec, Canada; Medical College of Wisconsin, Milwaukee, Wisconsin, USA

**Keywords:** therapeutic failure, immunocompromised host, few-colony inoculum population analysis minority-resistant subpopulations CLSI/EUCAST reference, heteroresistance, antimicrobial susceptibility testing

## Abstract

Conventional antimicrobial susceptibility testing (AST) is based on inocula derived from a small number of colonies, creating a substantial sampling bottleneck relative to the complexity of within-host bacterial populations. Although this limitation is well recognized, its clinical impact is likely limited in many immunocompetent hosts, where effective immune responses and adequate antimicrobial exposure buffer against the influence of low-frequency resistant subpopulations. However, genomic and mechanistic studies show that such subpopulations can exist at low frequencies and may become clinically relevant in high-risk settings, including persistent or high-burden infections and in profoundly immunocompromised patients. This article outlines the biological basis of the sampling constraint, clarifies when it is most likely to matter, and discusses targeted population-based and sequencing-informed adjunct approaches that may complement reference AST in select scenarios. Improving transparency around these limitations—without undermining confidence in standardized methods—supports antimicrobial stewardship and more nuanced interpretation in defined clinical contexts.

## COMMENTARY

Conventional antimicrobial susceptibility testing (AST) relies on inocula prepared from a small number of morphologically representative colonies, as specified in international reference standards such as International Organization for Standardization 20776-1 and the Clinical and Laboratory Standards Institute (CLSI) M100 (1, 2). This practice samples the subpopulation recovered from the clinical specimen and able to grow under laboratory culture conditions, rather than a direct census of the full within-host population at the infection site; as a result, it cannot systematically capture within-host diversity, including heteroresistance and polyclonality documented in genomic and mechanistic studies (3–6). Although the clinical significance of minority-resistant subpopulations varies by organism, host, and infection type, their existence is well established, and their potential to influence treatment response—particularly in high-risk contexts—warrants careful consideration.

Experimental and theoretical work in microbial evolution and population biology demonstrates that resistant variants can arise repeatedly and persist at low frequencies within large bacterial populations and can expand rapidly under antibiotic selection (3, 7). Within-host genomic studies further show that infections may contain multiple coexisting lineages and substantial intrahost diversity, including minority variants with resistance-associated genotypes (5, 8, 9). Together, these findings reinforce that “one isolate” may not fully represent the diversity present *in vivo*, particularly in chronic, high-burden, or persistent infections where population structure and spatial niches may permit diversification and coexistence (8, 9).

A clinically familiar analog is inducible AmpC-producing Enterobacterales, in which spontaneous mutations can be selected during therapy and lead to stable derepression of AmpC β-lactamase—most commonly described in *Citrobacter freundii* complex, *Enterobacter cloacae* complex, and *Klebsiella aerogenes*—resulting in the emergence of third-generation cephalosporin resistance within days after therapy initiation. In this setting, repeat culture and susceptibility testing of subsequent isolates may be warranted when clinically indicated (2, 10).

Heteroresistance further illustrates the limitations of few-colony sampling. Heteroresistance—defined as the presence of resistant subpopulations within an isolate otherwise classified as susceptible—can arise through diverse mechanisms, including gene amplification and unstable phenotypic states (3, 4, 11). In this commentary, we use “minority resistance” as an umbrella term for any low-frequency resistant subpopulation within the infecting population (including heteroresistance and polyclonal infections), whereas “heteroresistance” refers specifically to resistant subpopulations within a lineage otherwise categorized as susceptible. Heteroresistance has been described across multiple clinically important pathogens, including the ESKAPE group (*Enterococcus faecium*, *Staphylococcus aureus*, *Klebsiella pneumoniae*, *Acinetobacter baumannii*, *Pseudomonas aeruginosa*, and Enterobacterales), and its detection by routine AST is often inconsistent because minor subpopulations may be absent from the small founder set used to prepare inocula (12). Systematic reviews and clinical microbiology studies highlight that heteroresistance can be clinically relevant in selected settings and may contribute to discordant testing or therapeutic failure when minority populations expand during therapy (13, 14).

Although conventional AST performs well for the vast majority of infections—especially in immunocompetent hosts and in settings dominated by highly susceptible organisms—the reliance on a few selected colonies creates an inherent disconnect between the biological complexity of some infections and the analytical framework used to characterize susceptibility. Mechanistic and genomic studies collectively support that within-host populations may contain multiple coexisting lineages with distinct phenotypes, including minority-resistant subpopulations that can persist and expand under selective pressure (3–5, 8–14). Recognizing this gap does not diminish the value of current AST but highlights the need to clarify when sampling limitations are most likely to matter and how adjunct approaches might be applied selectively.

Clinical relevance is strongly context dependent. Minority resistance—whether manifest as heteroresistance or polyclonality—is most likely to influence outcomes in settings characterized by high bacterial burden, prolonged infection, limited source control, impaired host defenses (e.g., profound neutropenia, solid-organ transplantation), or constrained antibiotic penetration (15–17). For example, in a retrospective cohort of 255 patients with *Escherichia coli* bloodstream infection, heteroresistance prevalence varied substantially by agent (e.g., 43% for gentamicin and 9% for piperacillin–tazobactam, <1% for cefotaxime), was frequently misclassified as susceptible by routine testing, and was associated with adverse outcomes among patients treated with the corresponding antibiotic (8). Persistent *Staphylococcus aureus* infection, including phenotypes such as heteroresistant vancomycin-intermediate *S. aureus* (hVISA), illustrates how within-host diversity and resistance emergence can complicate therapy and interpretation, particularly when eradication depends heavily on antimicrobial activity (15, 17). More broadly, work on within-host pathogen population dynamics supports that persistence can provide the ecological time and structure needed for minority variants to survive and expand (18).

The continued reliance on few-colony inocula reflects the need for historical comparability, standardization, and regulatory reproducibility rather than biological representativeness. Guidance on AST practice and interpretation emphasizes that performance metrics such as categorical agreement, essential agreement, and very major error rates are anchored to the same standardized reference framework (19, 20). As a result, methods that more fully interrogate population diversity may yield discordant MICs despite capturing biologically meaningful resistance, complicating both regulatory evaluation and clinical interpretation. Recent clinical laboratory discussions have highlighted these limitations and the challenges of integrating heteroresistance-aware approaches into routine workflows (11–13).

Adjunct methodologies to mitigate the sampling bottleneck can be conceptualized as (i) increasing the breadth of phenotypic sampling and (ii) directly interrogating population structure by molecular means. Population-based phenotypic strategies include multi-colony sampling (testing multiple colonies with shared morphology), inocula prepared from colony sweeps, and selective or breakpoint screening plates to enrich detection of resistant subpopulations. In specific contexts, population analysis profiling remains a reference approach for characterizing heteroresistance (e.g., hVISA) and related phenotypes; simplified screen-agar strategies have been described to improve feasibility in clinical laboratories (21). Sequencing-informed approaches—including targeted resistance gene panels and next-generation sequencing strategies such as multi-colony whole-genome sequencing or deep sequencing of pooled colonies—may help detect mixed lineages or low-frequency resistance determinants but require careful interpretation because detection thresholds vary and genotype–phenotype relationships are incomplete for some organism–drug combinations (5, 8, 9). These adjunct approaches can increase detection probability but are constrained by workflow, contamination risk, turnaround time, cost, and interpretive complexity; accordingly, many remain confined to research or specialized settings. Their greatest value likely lies in targeted application to predefined high-risk scenarios rather than routine use (22).

Given these considerations, increased transparency regarding sampling constraints may benefit clinicians when applied judiciously. However, broadly worded statements in a patient chart carry important risks, including undermining confidence in validated AST methods and unintentionally driving broader antibiotic use. We therefore do not advocate a universal reporting statement. If local stakeholders elect to deploy an interpretive comment, it should be optional, restricted to predefined high-risk contexts and/or triggered by adjunct testing, and paired with practical clinical guidance aligned with antimicrobial stewardship. For example, “Susceptibility testing is performed on a limited number of colonies selected from culture, clinically relevant, low-frequency resistant subpopulations may not be detected. If clinical response is suboptimal, reassess source control and antimicrobial exposure (including dose optimization and site penetration), and consider repeat cultures and susceptibility testing and/or additional targeted testing in consultation with Infectious Diseases, Antimicrobial Stewardship, and the microbiology laboratory.”

Importantly, this proposed language reflects a pragmatic reporting strategy rather than a recommendation supported by direct outcome-based evidence for routine use, underscoring the need for prospective evaluation. In our view, when restricted to predefined high-risk contexts and linked to specific actions (reassessment, repeat cultures/AST when indicated, and stewardship/ID consultation), such comments may support informed decision-making while minimizing the risk of indiscriminate escalation of therapy.

Addressing minority-resistant subpopulations will require coordinated efforts that preserve confidence in standardized AST while providing clearer guidance on when its sampling limitations matter most. First, organism- and host-specific prospective studies are needed to define which patient populations are most affected and under what conditions minority resistance becomes clinically actionable (8, 9, 15–18). Second, adjunct population-based and molecular approaches should be integrated within established antimicrobial stewardship frameworks to ensure appropriate interpretation and avoid indiscriminate escalation. Third, laboratories, professional societies, and regulatory agencies should collaborate to develop evaluation pathways for population-aware methods that maintain continuity with reference AST while capturing clinically meaningful diversity (19–22). Finally, funding mechanisms should be explicitly planned: antimicrobial drug development programs and clinical trials could incorporate isolate banking and prespecified microbiological substudies (including multi-colony or sequencing-based analyses) to define minority-resistance prevalence and outcomes alongside traditional endpoints.
